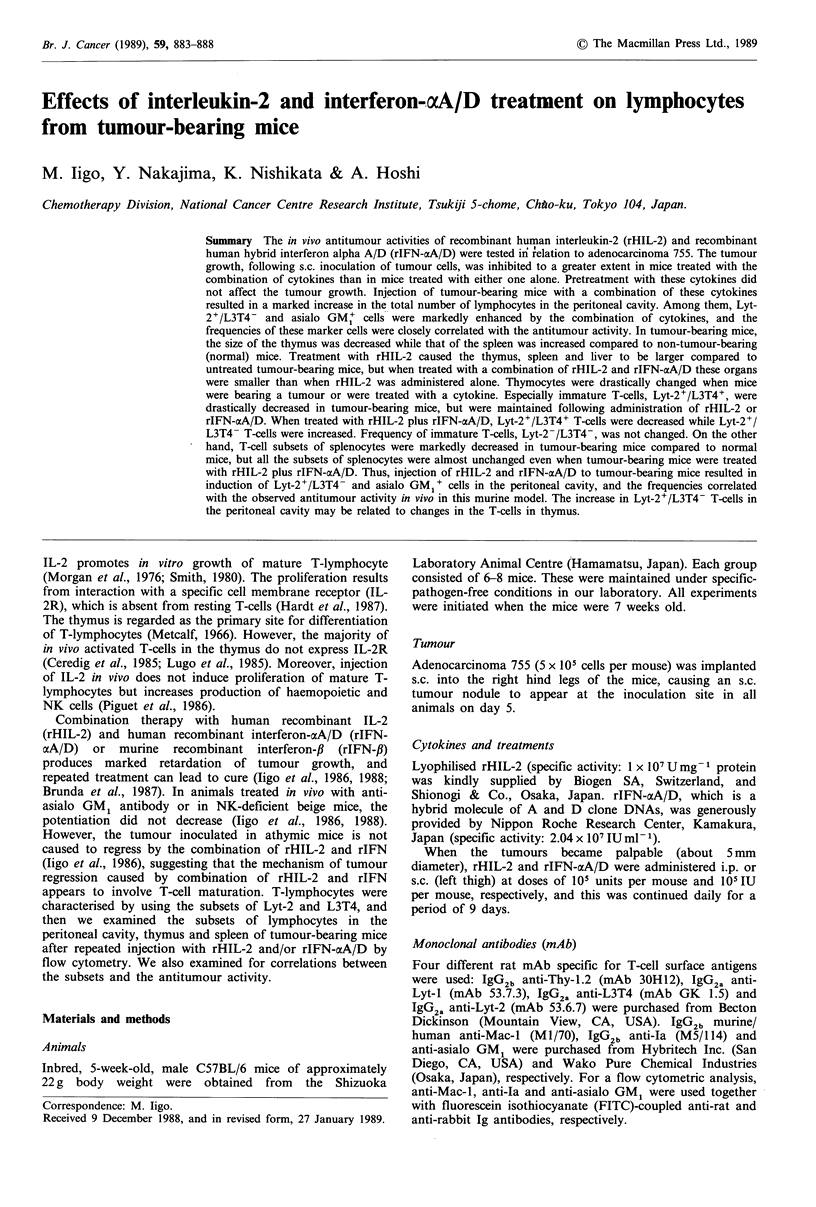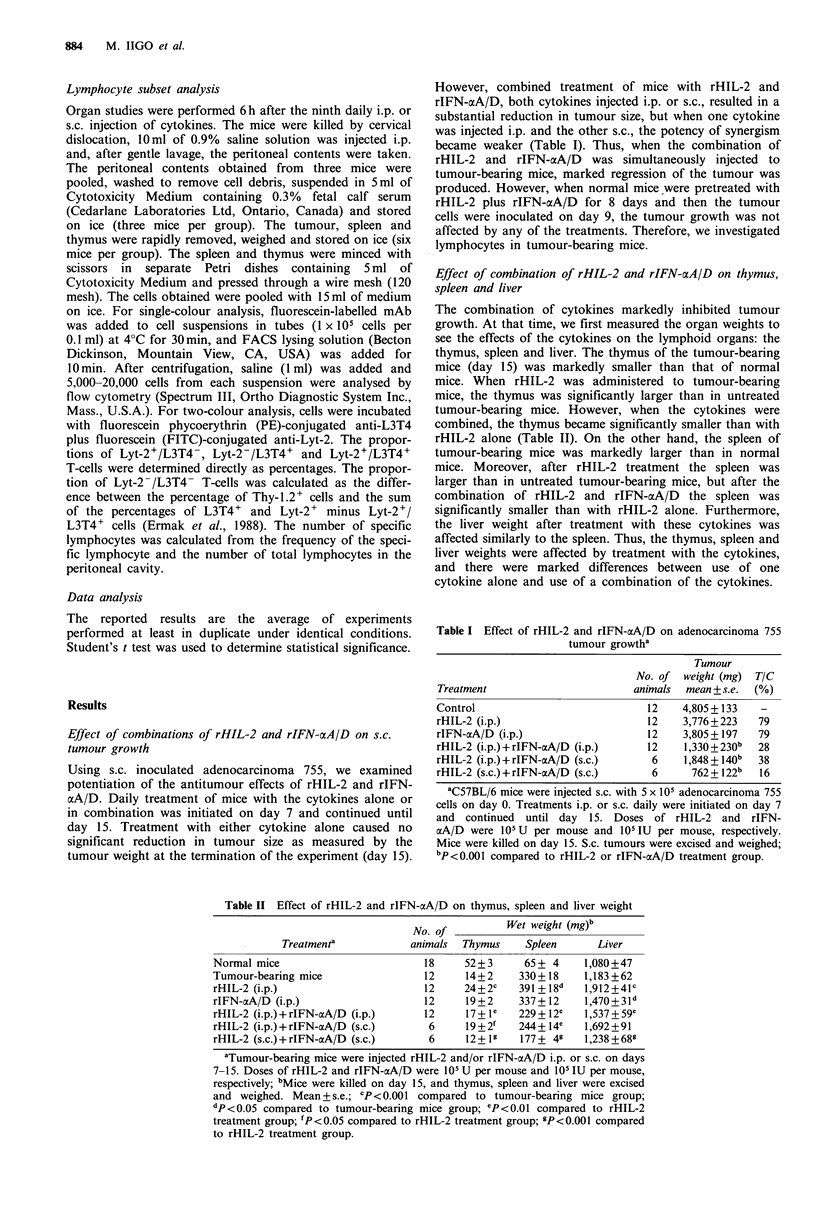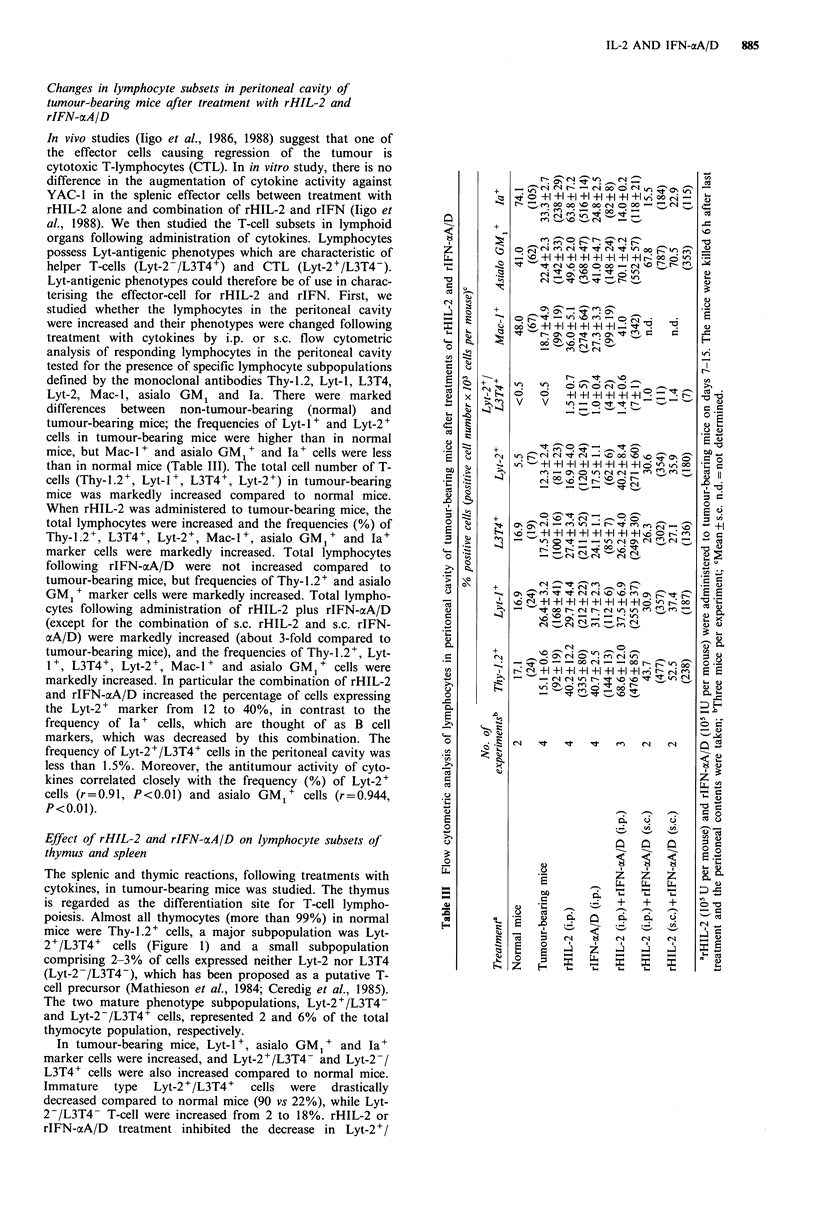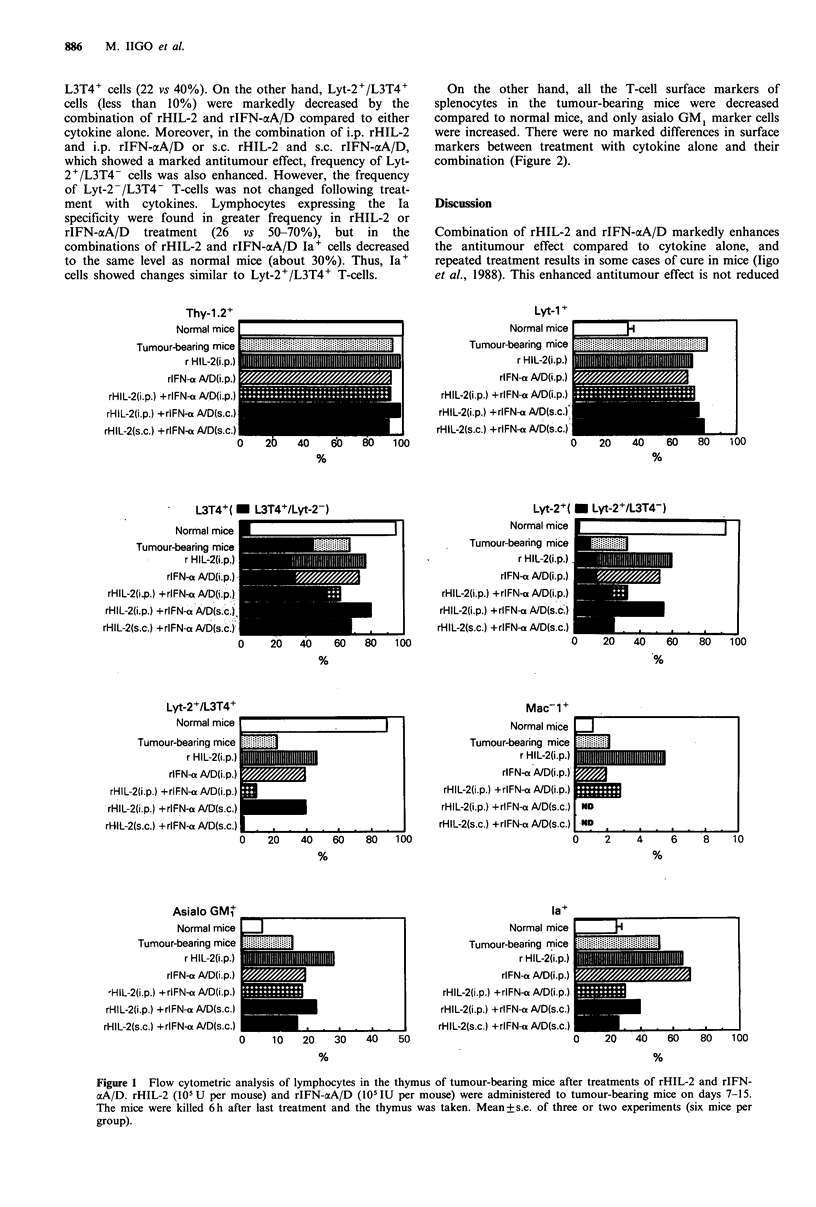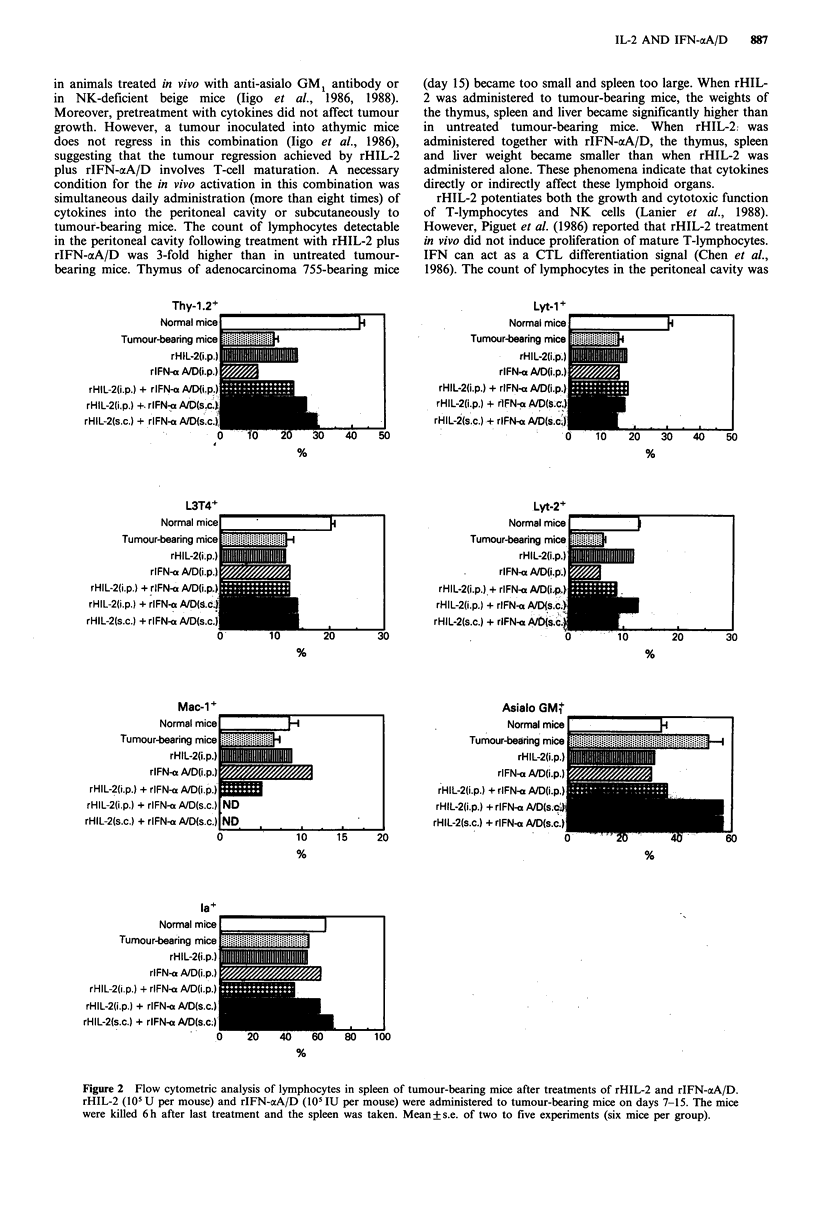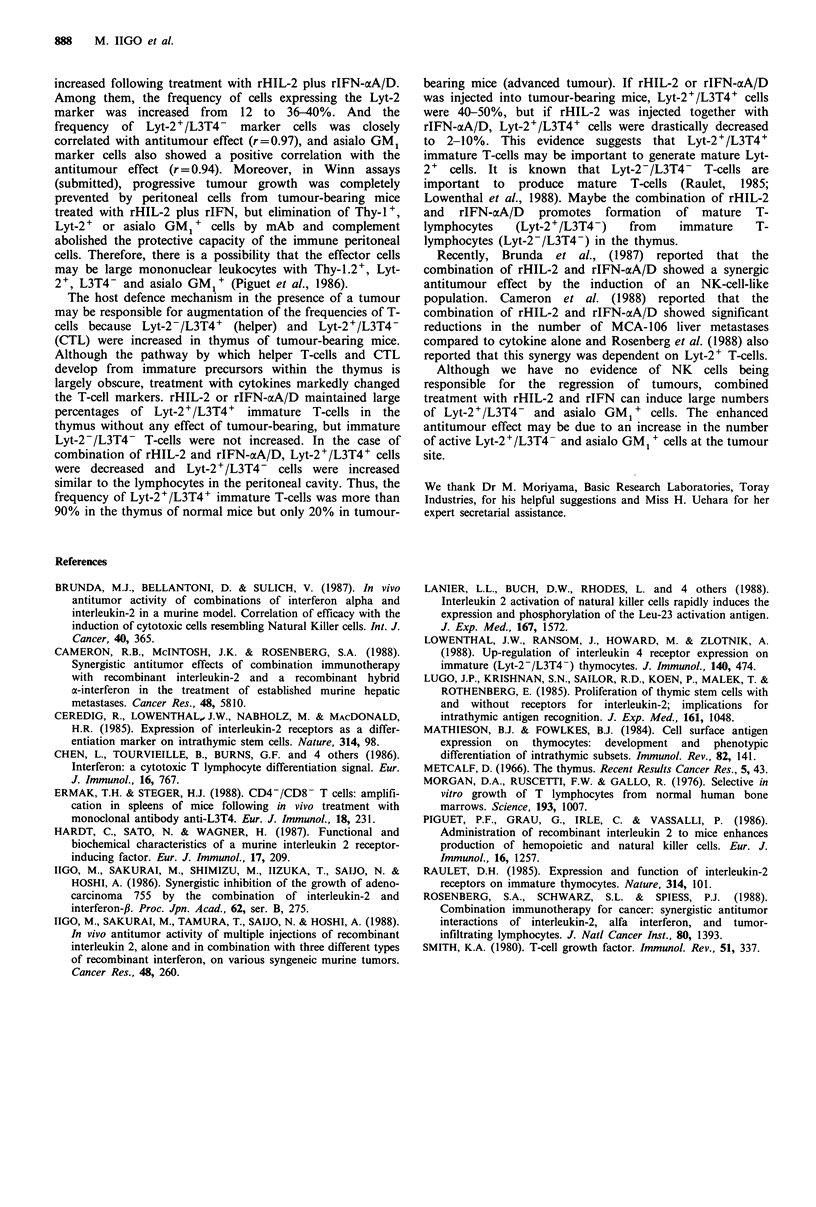# Effects of interleukin-2 and interferon-alpha A/D treatment on lymphocytes from tumour-bearing mice.

**DOI:** 10.1038/bjc.1989.187

**Published:** 1989-06

**Authors:** M. Ligo, Y. Nakajima, K. Nishikata, A. Hoshi

**Affiliations:** Chemotherapy Division, National Cancer Centre Research Institute, Tokyo, Japan.

## Abstract

The in vivo antitumour activities of recombinant human interleukin-2 (rHIL-2) and recombinant human hybrid interferon alpha A/D (rIFN-alpha A/D) were tested in relation to adenocarcinoma 755. The tumour growth, following s.c. inoculation of tumour cells, was inhibited to a greater extent in mice treated with the combination of cytokines than in mice treated with either one alone. Pretreatment with these cytokines did not affect the tumour growth. Injection of tumour-bearing mice with a combination of these cytokines resulted in a marked increase in the total number of lymphocytes in the peritoneal cavity. Among them, Lyt-2+/L3T4- and asialo GM1+ cells were markedly enhanced by the combination of cytokines, and the frequencies of these marker cells were closely correlated with the antitumour activity. In tumour-bearing mice, the size of the thymus was decreased while that of the spleen was increased compared to non-tumour-bearing (normal) mice. Treatment with rHIL-2 caused the thymus, spleen and liver to be larger compared to untreated tumour-bearing mice, but when treated with a combination of rHIL-2 and rIFN-alpha A/D these organs were smaller than when rHIL-2 was administered alone. Thymocytes were drastically changed when mice were bearing a tumour or were treated with a cytokine. Especially immature T-cells, Lyt-2+/L3T4+, were drastically decreased in tumour-bearing mice, but were maintained following administration of rHIL-2 or rIFN-alpha A/D. When treated with rHIL-2 plus rIFN-alpha A/D, Lyt-2+/L3T4+ T-cells were decreased while Lyt-2+/L3T4- T-cells were increased. Frequency of immature T-cells, Lyt-2-/L3T4-, was not changed. On the other hand, T-cell subsets of splenocytes were markedly decreased in tumour-bearing mice compared to normal mice, but all the subsets of splenocytes were almost unchanged even when tumour-bearing mice were treated with rHIL-2 plus rIFN-alpha A/D. Thus, injection of rHIL-2 and rIFN-alpha A/D to tumour-bearing mice resulted in induction of Lyt-2+/L3T4- and asialo GM1+ cells in the peritoneal cavity, and the frequencies correlated with the observed antitumour activity in vivo in this murine model. The increase in Lyt-2+/L3T4- T-cells in the peritoneal cavity may be related to changes in the T-cells in thymus.


*Br. J. Cancer* (1989), **59**, 883–888 © The Macmillan Press Ltd., 1989

### Effects of interleukin-2 and interferon-αA/D treatment on lymphocytes from tumour-bearing mice

M. Iigo, Y. Nakajima, K. Nishikata & A. Hoshi

*Chemotherapy Division, National Cancer Centre Research Institute, Tsukiji 5-chome, Chūo-ku, Tokyo 104, Japan.*

**Summary** The *in vivo* antitumour activities of recombinant human interleukin-2 (rHIL-2) and recombinant human hybrid interferon alpha A/D (rIFN-αA/D) were tested in relation to adenocarcinoma 755. The tumour growth, following s.c. inoculation of tumour cells, was inhibited to a greater extent in mice treated with the combination of cytokines than in mice treated with either one alone. Pretreatment with these cytokines did not affect the tumour growth. Injection of tumour-bearing mice with a combination of these cytokines resulted in a marked increase in the total number of lymphocytes in the peritoneal cavity. Among them, Lyt-$2^+$/$L3T4^-$ and asialo $GM_1^+$ cells were markedly enhanced by the combination of cytokines, and the frequencies of these marker cells were closely correlated with the antitumour activity. In tumour-bearing mice, the size of the thymus was decreased while that of the spleen was increased compared to non-tumour-bearing (normal) mice. Treatment with rHIL-2 caused the thymus, spleen and liver to be larger compared to untreated tumour-bearing mice, but when treated with a combination of rHIL-2 and rIFN-αA/D these organs were smaller than when rHIL-2 was administered alone. Thymocytes were drastically changed when mice were bearing a tumour or were treated with a cytokine. Especially immature T-cells, Lyt-$2^+$/$L3T4^+$, were drastically decreased in tumour-bearing mice, but were maintained following administration of rHIL-2 or rIFN-αA/D. When treated with rHIL-2 plus rIFN-αA/D, Lyt-$2^+$/$L3T4^+$ T-cells were decreased while Lyt-$2^+$/$L3T4^-$ T-cells were increased. Frequency of immature T-cells, Lyt-$2^-$/$L3T4^-$, was not changed. On the other hand, T-cell subsets of splenocytes were markedly decreased in tumour-bearing mice compared to normal mice, but all the subsets of splenocytes were almost unchanged even when tumour-bearing mice were treated with rHIL-2 plus rIFN-αA/D. Thus, injection of rHIL-2 and rIFN-αA/D to tumour-bearing mice resulted in induction of Lyt-$2^+$/$L3T4^-$ and asialo $GM_1{}^+$ cells in the peritoneal cavity, and the frequencies correlated with the observed antitumour activity *in vivo* in this murine model. The increase in Lyt-$2^+$/$L3T4^-$ T-cells in the peritoneal cavity may be related to changes in the T-cells in thymus.

IL-2 promotes *in vitro* growth of mature T-lymphocyte (Morgan *et al.*, 1976; Smith, 1980). The proliferation results from interaction with a specific cell membrane receptor (IL-2R), which is absent from resting T-cells (Hardt *et al.*, 1987). The thymus is regarded as the primary site for differentiation of T-lymphocytes (Metcalf, 1966). However, the majority of *in vivo* activated T-cells in the thymus do not express IL-2R (Ceredig *et al.*, 1985; Lugo *et al.*, 1985). Moreover, injection of IL-2 *in vivo* does not induce proliferation of mature T-lymphocytes but increases production of haemopoietic and NK cells (Piguet *et al.*, 1986).

Combination therapy with human recombinant IL-2 (rHIL-2) and human recombinant interferon-αA/D (rIFN-αA/D) or murine recombinant interferon-β (rIFN-β) produces marked retardation of tumour growth, and repeated treatment can lead to cure (Iigo *et al.*, 1986, 1988; Brunda *et al.*, 1987). In animals treated *in vivo* with anti-asialo $GM_1$ antibody or in NK-deficient beige mice, the potentiation did not decrease (Iigo *et al.*, 1986, 1988). However, the tumour inoculated in athymic mice is not caused to regress by the combination of rHIL-2 and rIFN (Iigo *et al.*, 1986), suggesting that the mechanism of tumour regression caused by combination of rHIL-2 and rIFN appears to involve T-cell maturation. T-lymphocytes were characterised by using the subsets of Lyt-2 and L3T4, and then we examined the subsets of lymphocytes in the peritoneal cavity, thymus and spleen of tumour-bearing mice after repeated injection with rHIL-2 and/or rIFN-αA/D by flow cytometry. We also examined for correlations between the subsets and the antitumour activity.

### Materials and methods

### *Animals*

Inbred, 5-week-old, male C57BL/6 mice of approximately 22 g body weight were obtained from the Shizuoka Laboratory Animal Centre (Hamamatsu, Japan). Each group consisted of 6–8 mice. These were maintained under specific-pathogen-free conditions in our laboratory. All experiments were initiated when the mice were 7 weeks old.

### *Tumour*

Adenocarcinoma 755 ($5 \times 10^5$ cells per mouse) was implanted s.c. into the right hind legs of the mice, causing an s.c. tumour nodule to appear at the inoculation site in all animals on day 5.

### *Cytokines and treatments*

Lyophilised rHIL-2 (specific activity: $1 \times 10^7$ U mg$^{-1}$ protein was kindly supplied by Biogen SA, Switzerland, and Shionogi & Co., Osaka, Japan. rIFN-αA/D, which is a hybrid molecule of A and D clone DNAs, was generously provided by Nippon Roche Research Center, Kamakura, Japan (specific activity: $2.04 \times 10^7$ IU ml$^{-1}$).

When the tumours became palpable (about 5 mm diameter), rHIL-2 and rIFN-αA/D were administered i.p. or s.c. (left thigh) at doses of $10^5$ units per mouse and $10^5$ IU per mouse, respectively, and this was continued daily for a period of 9 days.

### *Monoclonal antibodies (mAb)*

Four different rat mAb specific for T-cell surface antigens were used: $IgG_{2b}$ anti-Thy-1.2 (mAb 30H12), $IgG_{2a}$ anti-Lyt-1 (mAb 53.7.3), $IgG_{2a}$ anti-L3T4 (mAb GK 1.5) and $IgG_{2a}$ anti-Lyt-2 (mAb 53.6.7) were purchased from Becton Dickinson (Mountain View, CA, USA). $IgG_{2b}$ murine/human anti-Mac-1 (M1/70), $IgG_{2b}$ anti-Ia (M5/114) and anti-asialo $GM_1$ were purchased from Hybritech Inc. (San Diego, CA, USA) and Wako Pure Chemical Industries (Osaka, Japan), respectively. For a flow cytometric analysis, anti-Mac-1, anti-Ia and anti-asialo $GM_1$ were used together with fluorescein isothiocyanate (FITC)-coupled anti-rat and anti-rabbit Ig antibodies, respectively.

Correspondence: M. Iigo.
Received 9 December 1988, and in revised form, 27 January 1989.

*Lymphocyte subset analysis*

Organ studies were performed 6 h after the ninth daily i.p. or s.c. injection of cytokines. The mice were killed by cervical dislocation, 10 ml of 0.9% saline solution was injected i.p. and, after gentle lavage, the peritoneal contents were taken. The peritoneal contents obtained from three mice were pooled, washed to remove cell debris, suspended in 5 ml of Cytotoxicity Medium containing 0.3% fetal calf serum (Cedarlane Laboratories Ltd, Ontario, Canada) and stored on ice (three mice per group). The tumour, spleen and thymus were rapidly removed, weighed and stored on ice (six mice per group). The spleen and thymus were minced with scissors in separate Petri dishes containing 5 ml of Cytotoxicity Medium and pressed through a wire mesh (120 mesh). The cells obtained were pooled with 15 ml of medium on ice. For single-colour analysis, fluorescein-labelled mAb was added to cell suspensions in tubes ($1 \times 10^5$ cells per 0.1 ml) at 4°C for 30 min, and FACS lysing solution (Becton Dickinson, Mountain View, CA, USA) was added for 10 min. After centrifugation, saline (1 ml) was added and 5,000–20,000 cells from each suspension were analysed by flow cytometry (Spectrum III, Ortho Diagnostic System Inc., Mass., U.S.A.). For two-colour analysis, cells were incubated with fluorescein phycoerythrin (PE)-conjugated anti-L3T4 plus fluorescein (FITC)-conjugated anti-Lyt-2. The proportions of $Lyt\text{-}2^+/L3T4^-$, $Lyt\text{-}2^-/L3T4^+$ and $Lyt\text{-}2^+/L3T4^+$ T-cells were determined directly as percentages. The proportion of $Lyt\text{-}2^-/L3T4^-$ T-cells was calculated as the difference between the percentage of $Thy\text{-}1.2^+$ cells and the sum of the percentages of $L3T4^+$ and $Lyt\text{-}2^+$ minus $Lyt\text{-}2^+/L3T4^+$ cells (Ermak *et al.*, 1988). The number of specific lymphocytes was calculated from the frequency of the specific lymphocyte and the number of total lymphocytes in the peritoneal cavity.

*Data analysis*

The reported results are the average of experiments performed at least in duplicate under identical conditions. Student's *t* test was used to determine statistical significance.

### Results

*Effect of combinations of rHIL-2 and rIFN-αA/D on s.c. tumour growth*

Using s.c. inoculated adenocarcinoma 755, we examined potentiation of the antitumour effects of rHIL-2 and rIFN-αA/D. Daily treatment of mice with the cytokines alone or in combination was initiated on day 7 and continued until day 15. Treatment with either cytokine alone caused no significant reduction in tumour size as measured by the tumour weight at the termination of the experiment (day 15). However, combined treatment of mice with rHIL-2 and rIFN-αA/D, both cytokines injected i.p. or s.c., resulted in a substantial reduction in tumour size, but when one cytokine was injected i.p. and the other s.c., the potency of synergism became weaker (Table I). Thus, when the combination of rHIL-2 and rIFN-αA/D was simultaneously injected to tumour-bearing mice, marked regression of the tumour was produced. However, when normal mice were pretreated with rHIL-2 plus rIFN-αA/D for 8 days and then the tumour cells were inoculated on day 9, the tumour growth was not affected by any of the treatments. Therefore, we investigated lymphocytes in tumour-bearing mice.

*Effect of combination of rHIL-2 and rIFN-αA/D on thymus, spleen and liver*

The combination of cytokines markedly inhibited tumour growth. At that time, we first measured the organ weights to see the effects of the cytokines on the lymphoid organs: the thymus, spleen and liver. The thymus of the tumour-bearing mice (day 15) was markedly smaller than that of normal mice. When rHIL-2 was administered to tumour-bearing mice, the thymus was significantly larger than in untreated tumour-bearing mice. However, when the cytokines were combined, the thymus became significantly smaller than with rHIL-2 alone (Table II). On the other hand, the spleen of tumour-bearing mice was markedly larger than in normal mice. Moreover, after rHIL-2 treatment the spleen was larger than in untreated tumour-bearing mice, but after the combination of rHIL-2 and rIFN-αA/D the spleen was significantly smaller than with rHIL-2 alone. Furthermore, the liver weight after treatment with these cytokines was affected similarly to the spleen. Thus, the thymus, spleen and liver weights were affected by treatment with the cytokines, and there were marked differences between use of one cytokine alone and use of a combination of the cytokines.

**Table I** Effect of rHIL-2 and rIFN-αA/D on adenocarcinoma 755 tumour growth[a]

| *Treatment* | *No. of animals* | *Tumour weight (mg) mean ± s.e.* | *T/C (%)* |
|---|---|---|---|
| Control | 12 | 4,805 ± 133 | – |
| rHIL-2 (i.p.) | 12 | 3,776 ± 223 | 79 |
| rIFN-αA/D (i.p.) | 12 | 3,805 ± 197 | 79 |
| rHIL-2 (i.p.) + rIFN-αA/D (i.p.) | 12 | 1,330 ± 230[b] | 28 |
| rHIL-2 (i.p.) + rIFN-αA/D (s.c.) | 6 | 1,848 ± 140[b] | 38 |
| rHIL-2 (s.c.) + rIFN-αA/D (s.c.) | 6 | 762 ± 122[b] | 16 |

[a]C57BL/6 mice were injected s.c. with $5 \times 10^5$ adenocarcinoma 755 cells on day 0. Treatments i.p. or s.c. daily were initiated on day 7 and continued until day 15. Doses of rHIL-2 and rIFN-αA/D were $10^5$ U per mouse and $10^5$ IU per mouse, respectively. Mice were killed on day 15. S.c. tumours were excised and weighed; [b]$P < 0.001$ compared to rHIL-2 or rIFN-αA/D treatment group.

**Table II** Effect of rHIL-2 and rIFN-αA/D on thymus, spleen and liver weight

| *Treatment*[a] | *No. of animals* | *Wet weight (mg)*[b] *Thymus* | *Spleen* | *Liver* |
|---|---|---|---|---|
| Normal mice | 18 | 52 ± 3 | 65 ± 4 | 1,080 ± 47 |
| Tumour-bearing mice | 12 | 14 ± 2 | 330 ± 18 | 1,183 ± 62 |
| rHIL-2 (i.p.) | 12 | 24 ± 2[c] | 391 ± 18[d] | 1,912 ± 41[c] |
| rIFN-αA/D (i.p.) | 12 | 19 ± 2 | 337 ± 12 | 1,470 ± 31[d] |
| rHIL-2 (i.p.) + rIFN-αA/D (i.p.) | 12 | 17 ± 1[e] | 229 ± 12[e] | 1,537 ± 59[e] |
| rHIL-2 (i.p.) + rIFN-αA/D (s.c.) | 6 | 19 ± 2[f] | 244 ± 14[e] | 1,692 ± 91 |
| rHIL-2 (s.c.) + rIFN-αA/D (s.c.) | 6 | 12 ± 1[g] | 177 ± 4[g] | 1,238 ± 68[g] |

[a]Tumour-bearing mice were injected rHIL-2 and/or rIFN-αA/D i.p. or s.c. on days 7–15. Doses of rHIL-2 and rIFN-αA/D were $10^5$ U per mouse and $10^5$ IU per mouse, respectively; [b]Mice were killed on day 15, and thymus, spleen and liver were excised and weighed. Mean ± s.e.; [c]$P < 0.001$ compared to tumour-bearing mice group; [d]$P < 0.05$ compared to tumour-bearing mice group; [e]$P < 0.01$ compared to rHIL-2 treatment group; [f]$P < 0.05$ compared to rHIL-2 treatment group; [g]$P < 0.001$ compared to rHIL-2 treatment group.

*Changes in lymphocyte subsets in peritoneal cavity of tumour-bearing mice after treatment with rHIL-2 and rIFN-αA/D*

*In vivo* studies (Iigo *et al.*, 1986, 1988) suggest that one of the effector cells causing regression of the tumour is cytotoxic T-lymphocytes (CTL). In *in vitro* study, there is no difference in the augmentation of cytokine activity against YAC-1 in the splenic effector cells between treatment with rHIL-2 alone and combination of rHIL-2 and rIFN (Iigo *et al.*, 1988). We then studied the T-cell subsets in lymphoid organs following administration of cytokines. Lymphocytes possess Lyt-antigenic phenotypes which are characteristic of helper T-cells (Lyt-2$^-$/L3T4$^+$) and CTL (Lyt-2$^+$/L3T4$^-$). Lyt-antigenic phenotypes could therefore be of use in characterising the effector-cell for rHIL-2 and rIFN. First, we studied whether the lymphocytes in the peritoneal cavity were increased and their phenotypes were changed following treatment with cytokines by i.p. or s.c. flow cytometric analysis of responding lymphocytes in the peritoneal cavity tested for the presence of specific lymphocyte subpopulations defined by the monoclonal antibodies Thy-1.2, Lyt-1, L3T4, Lyt-2, Mac-1, asialo $GM_1$ and Ia. There were marked differences between non-tumour-bearing (normal) and tumour-bearing mice; the frequencies of Lyt-1$^+$ and Lyt-2$^+$ cells in tumour-bearing mice were higher than in normal mice, but Mac-1$^+$ and asialo $GM_1{}^+$ and Ia$^+$ cells were less than in normal mice (Table III). The total cell number of T-cells (Thy-1.2$^+$, Lyt-1$^+$, L3T4$^+$, Lyt-2$^+$) in tumour-bearing mice was markedly increased compared to normal mice. When rHIL-2 was administered to tumour-bearing mice, the total lymphocytes were increased and the frequencies (%) of Thy-1.2$^+$, L3T4$^+$, Lyt-2$^+$, Mac-1$^+$, asialo $GM_1{}^+$ and Ia$^+$ marker cells were markedly increased. Total lymphocytes following rIFN-αA/D were not increased compared to tumour-bearing mice, but frequencies of Thy-1.2$^+$ and asialo $GM_1{}^+$ marker cells were markedly increased. Total lymphocytes following administration of rHIL-2 plus rIFN-αA/D (except for the combination of s.c. rHIL-2 and s.c. rIFN-αA/D) were markedly increased (about 3-fold compared to tumour-bearing mice), and the frequencies of Thy-1.2$^+$, Lyt-1$^+$, L3T4$^+$, Lyt-2$^+$, Mac-1$^+$ and asialo $GM_1{}^+$ cells were markedly increased. In particular the combination of rHIL-2 and rIFN-αA/D increased the percentage of cells expressing the Lyt-2$^+$ marker from 12 to 40%, in contrast to the frequency of Ia$^+$ cells, which are thought of as B cell markers, which was decreased by this combination. The frequency of Lyt-2$^+$/L3T4$^+$ cells in the peritoneal cavity was less than 1.5%. Moreover, the antitumour activity of cytokines correlated closely with the frequency (%) of Lyt-2$^+$ cells ($r$=0.91, $P$<0.01) and asialo $GM_1{}^+$ cells ($r$=0.944, $P$<0.01).

*Effect of rHIL-2 and rIFN-αA/D on lymphocyte subsets of thymus and spleen*

The splenic and thymic reactions, following treatments with cytokines, in tumour-bearing mice was studied. The thymus is regarded as the differentiation site for T-cell lymphopoiesis. Almost all thymocytes (more than 99%) in normal mice were Thy-1.2$^+$ cells, a major subpopulation was Lyt-2$^+$/L3T4$^+$ cells (Figure 1) and a small subpopulation comprising 2–3% of cells expressed neither Lyt-2 nor L3T4 (Lyt-2$^-$/L3T4$^-$), which has been proposed as a putative T-cell precursor (Mathieson *et al.*, 1984; Ceredig *et al.*, 1985). The two mature phenotype subpopulations, Lyt-2$^+$/L3T4$^-$ and Lyt-2$^-$/L3T4$^+$ cells, represented 2 and 6% of the total thymocyte population, respectively.

In tumour-bearing mice, Lyt-1$^+$, asialo $GM_1{}^+$ and Ia$^+$ marker cells were increased, and Lyt-2$^+$/L3T4$^-$ and Lyt-2$^-$/L3T4$^+$ cells were also increased compared to normal mice. Immature type Lyt-2$^+$/L3T4$^+$ cells were drastically decreased compared to normal mice (90 *vs* 22%), while Lyt-2$^-$/L3T4$^-$ T-cell were increased from 2 to 18%. rHIL-2 or rIFN-αA/D treatment inhibited the decrease in Lyt-2$^+$/

**Table III** Flow cytometric analysis of lymphocytes in peritoneal cavity of tumour-bearing mice after treatments of rHIL-2 and rIFN-αA/D

| Treatment[a] | No. of experiments[b] | % positive cells (positive cell number × $10^3$ cells per mouse)[c] Thy-1.2$^+$ | Lyt-1$^+$ | L3T4$^+$ | Lyt-2$^+$ | Lyt-2$^+$/L3T4$^+$ | Mac-1$^+$ | Asialo $GM_1{}^+$ | Ia$^+$ |
|---|---|---|---|---|---|---|---|---|---|
| Normal mice | 2 | 17.1 (24) | 16.9 (24) | 16.9 (19) | 5.5 (7) | <0.5 | 48.0 (67) | 41.0 (62) | 74.1 (105) |
| Tumour-bearing mice | 4 | 15.1±0.6 (92±19) | 26.4±3.2 (168±41) | 17.5±2.0 (100±16) | 12.3±2.4 (81±23) | <0.5 | 18.7±4.9 (99±19) | 22.4±2.3 (142±33) | 33.3±2.7 (238±29) |
| rHIL-2 (i.p.) | 4 | 40.2±12.2 (335±80) | 29.7±4.4 (212±22) | 27.4±3.4 (211±52) | 16.9±4.0 (120±24) | 1.5±0.7 (11±5) | 36.0±5.1 (274±64) | 49.6±2.0 (368±47) | 63.8±7.2 (516±14) |
| rIFN-αA/D (i.p.) | 4 | 40.7±2.5 (144±13) | 31.7±2.3 (112±6) | 24.1±1.1 (85±7) | 17.5±1.1 (62±6) | 1.0±0.4 (4±2) | 27.3±3.3 (99±19) | 41.0±4.7 (148±24) | 24.8±2.5 (82±8) |
| rHIL-2 (i.p.)+rIFN-αA/D (i.p.) | 3 | 68.6±12.0 (476±85) | 37.5±6.9 (255±37) | 26.2±4.0 (249±30) | 40.2±8.4 (271±60) | 1.4±0.6 (7±1) | 41.0 (342) | 70.1±4.2 (552±57) | 14.0±0.2 (118±21) |
| rHIL-2 (i.p.)+rIFN-αA/D (s.c.) | 2 | 43.7 (477) | 30.9 (357) | 26.3 (302) | 30.6 (354) | 1.0 (11) | n.d. | 67.8 (787) | 15.5 (184) |
| rHIL-2 (s.c.)+rIFN-αA/D (s.c.) | 2 | 52.5 (238) | 37.4 (187) | 27.1 (136) | 35.9 (180) | 1.4 (7) | n.d. | 70.5 (353) | 22.9 (115) |

[a]rHIL-2 ($10^5$ U per mouse) and rIFN-αA/D ($10^5$ IU per mouse) were administered to tumour-bearing mice on days 7–15. The mice were killed 6 h after last treatment and the peritoneal contents were taken; [b]Three mice per experiment; [c]Mean±s.e. n.d.=not determined.

L3T4$^+$ cells (22 *vs* 40%). On the other hand, Lyt-2$^+$/L3T4$^+$ cells (less than 10%) were markedly decreased by the combination of rHIL-2 and rIFN-αA/D compared to either cytokine alone. Moreover, in the combination of i.p. rHIL-2 and i.p. rIFN-αA/D or s.c. rHIL-2 and s.c. rIFN-αA/D, which showed a marked antitumour effect, frequency of Lyt-2$^+$/L3T4$^-$ cells was also enhanced. However, the frequency of Lyt-2$^-$/L3T4$^-$ T-cells was not changed following treatment with cytokines. Lymphocytes expressing the Ia specificity were found in greater frequency in rHIL-2 or rIFN-αA/D treatment (26 *vs* 50–70%), but in the combinations of rHIL-2 and rIFN-αA/D Ia$^+$ cells decreased to the same level as normal mice (about 30%). Thus, Ia$^+$ cells showed changes similar to Lyt-2$^+$/L3T4$^+$ T-cells.

On the other hand, all the T-cell surface markers of splenocytes in the tumour-bearing mice were decreased compared to normal mice, and only asialo $GM_1$ marker cells were increased. There were no marked differences in surface markers between treatment with cytokine alone and their combination (Figure 2).

### Discussion

Combination of rHIL-2 and rIFN-αA/D markedly enhances the antitumour effect compared to cytokine alone, and repeated treatment results in some cases of cure in mice (Iigo *et al.*, 1988). This enhanced antitumour effect is not reduced

**Figure 1** Flow cytometric analysis of lymphocytes in the thymus of tumour-bearing mice after treatments of rHIL-2 and rIFN-αA/D. rHIL-2 ($10^5$ U per mouse) and rIFN-αA/D ($10^5$ IU per mouse) were administered to tumour-bearing mice on days 7–15. The mice were killed 6 h after last treatment and the thymus was taken. Mean ± s.e. of three or two experiments (six mice per group).

in animals treated *in vivo* with anti-asialo $GM_1$ antibody or in NK-deficient beige mice (Iigo *et al.*, 1986, 1988). Moreover, pretreatment with cytokines did not affect tumour growth. However, a tumour inoculated into athymic mice does not regress in this combination (Iigo *et al.*, 1986), suggesting that the tumour regression achieved by rHIL-2 plus rIFN-αA/D involves T-cell maturation. A necessary condition for the *in vivo* activation in this combination was simultaneous daily administration (more than eight times) of cytokines into the peritoneal cavity or subcutaneously to tumour-bearing mice. The count of lymphocytes detectable in the peritoneal cavity following treatment with rHIL-2 plus rIFN-αA/D was 3-fold higher than in untreated tumour-bearing mice. Thymus of adenocarcinoma 755-bearing mice (day 15) became too small and spleen too large. When rHIL-2 was administered to tumour-bearing mice, the weights of the thymus, spleen and liver became significantly higher than in untreated tumour-bearing mice. When rHIL-2 was administered together with rIFN-αA/D, the thymus, spleen and liver weight became smaller than when rHIL-2 was administered alone. These phenomena indicate that cytokines directly or indirectly affect these lymphoid organs.

rHIL-2 potentiates both the growth and cytotoxic function of T-lymphocytes and NK cells (Lanier *et al.*, 1988). However, Piguet *et al.* (1986) reported that rHIL-2 treatment *in vivo* did not induce proliferation of mature T-lymphocytes. IFN can act as a CTL differentiation signal (Chen *et al.*, 1986). The count of lymphocytes in the peritoneal cavity was

**Figure 2** Flow cytometric analysis of lymphocytes in spleen of tumour-bearing mice after treatments of rHIL-2 and rIFN-αA/D. rHIL-2 ($10^5$ U per mouse) and rIFN-αA/D ($10^5$ IU per mouse) were administered to tumour-bearing mice on days 7–15. The mice were killed 6 h after last treatment and the spleen was taken. Mean ± s.e. of two to five experiments (six mice per group).

increased following treatment with rHIL-2 plus rIFN-αA/D. Among them, the frequency of cells expressing the Lyt-2 marker was increased from 12 to 36–40%. And the frequency of Lyt-$2^+$/L3T$4^-$ marker cells was closely correlated with antitumour effect ($r=0.97$), and asialo $GM_1$ marker cells also showed a positive correlation with the antitumour effect ($r=0.94$). Moreover, in Winn assays (submitted), progressive tumour growth was completely prevented by peritoneal cells from tumour-bearing mice treated with rHIL-2 plus rIFN, but elimination of Thy-$1^+$, Lyt-$2^+$ or asialo $GM_1{}^+$ cells by mAb and complement abolished the protective capacity of the immune peritoneal cells. Therefore, there is a possibility that the effector cells may be large mononuclear leukocytes with Thy-$1.2^+$, Lyt-$2^+$, L3T$4^-$ and asialo $GM_1{}^+$ (Piguet et al., 1986).

The host defence mechanism in the presence of a tumour may be responsible for augmentation of the frequencies of T-cells because Lyt-$2^-$/L3T$4^+$ (helper) and Lyt-$2^+$/L3T$4^-$ (CTL) were increased in thymus of tumour-bearing mice. Although the pathway by which helper T-cells and CTL develop from immature precursors within the thymus is largely obscure, treatment with cytokines markedly changed the T-cell markers. rHIL-2 or rIFN-αA/D maintained large percentages of Lyt-$2^+$/L3T$4^+$ immature T-cells in the thymus without any effect of tumour-bearing, but immature Lyt-$2^-$/L3T$4^-$ T-cells were not increased. In the case of combination of rHIL-2 and rIFN-αA/D, Lyt-$2^+$/L3T$4^+$ cells were decreased and Lyt-$2^+$/L3T$4^-$ cells were increased similar to the lymphocytes in the peritoneal cavity. Thus, the frequency of Lyt-$2^+$/L3T$4^+$ immature T-cells was more than 90% in the thymus of normal mice but only 20% in tumour-bearing mice (advanced tumour). If rHIL-2 or rIFN-αA/D was injected into tumour-bearing mice, Lyt-$2^+$/L3T$4^+$ cells were 40–50%, but if rHIL-2 was injected together with rIFN-αA/D, Lyt-$2^+$/L3T$4^+$ cells were drastically decreased to 2–10%. This evidence suggests that Lyt-$2^+$/L3T$4^+$ immature T-cells may be important to generate mature Lyt-$2^+$ cells. It is known that Lyt-$2^-$/L3T$4^-$ T-cells are important to produce mature T-cells (Raulet, 1985; Lowenthal et al., 1988). Maybe the combination of rHIL-2 and rIFN-αA/D promotes formation of mature T-lymphocytes (Lyt-$2^+$/L3T$4^-$) from immature T-lymphocytes (Lyt-$2^-$/L3T$4^-$) in the thymus.

Recently, Brunda et al., (1987) reported that the combination of rHIL-2 and rIFN-αA/D showed a synergic antitumour effect by the induction of an NK-cell-like population. Cameron et al. (1988) reported that the combination of rHIL-2 and rIFN-αA/D showed significant reductions in the number of MCA-106 liver metastases compared to cytokine alone and Rosenberg et al. (1988) also reported that this synergy was dependent on Lyt-$2^+$ T-cells.

Although we have no evidence of NK cells being responsible for the regression of tumours, combined treatment with rHIL-2 and rIFN can induce large numbers of Lyt-$2^+$/L3T$4^-$ and asialo $GM_1{}^+$ cells. The enhanced antitumour effect may be due to an increase in the number of active Lyt-$2^+$/L3T$4^-$ and asialo $GM_1{}^+$ cells at the tumour site.

We thank Dr M. Moriyama, Basic Research Laboratories, Toray Industries, for his helpful suggestions and Miss H. Uehara for her expert secretarial assistance.

### References

BRUNDA, M.J., BELLANTONI, D. & SULICH, V. (1987). *In vivo* antitumor activity of combinations of interferon alpha and interleukin-2 in a murine model. Correlation of efficacy with the induction of cytotoxic cells resembling Natural Killer cells. *Int. J. Cancer*, **40**, 365.

CAMERON, R.B., McINTOSH, J.K. & ROSENBERG, S.A. (1988). Synergistic antitumor effects of combination immunotherapy with recombinant interleukin-2 and a recombinant hybrid α-interferon in the treatment of established murine hepatic metastases. *Cancer Res.*, **48**, 5810.

CEREDIG, R., LOWENTHAL, J.W., NABHOLZ, M. & MacDONALD, H.R. (1985). Expression of interleukin-2 receptors as a differentiation marker on intrathymic stem cells. *Nature*, **314**, 98.

CHEN, L., TOURVIEILLE, B., BURNS, G.F. and 4 others (1986). Interferon: a cytotoxic T lymphocyte differentiation signal. *Eur. J. Immunol.*, **16**, 767.

ERMAK, T.H. & STEGER, H.J. (1988). CD$4^-$/CD$8^-$ T cells: amplification in spleens of mice following *in vivo* treatment with monoclonal antibody anti-L3T4. *Eur. J. Immunol.*, **18**, 231.

HARDT, C., SATO, N. & WAGNER, H. (1987). Functional and biochemical characteristics of a murine interleukin 2 receptor-inducing factor. *Eur. J. Immunol.*, **17**, 209.

IIGO, M., SAKURAI, M., SHIMIZU, M., IIZUKA, T., SAIJO, N. & HOSHI, A. (1986). Synergistic inhibition of the growth of adenocarcinoma 755 by the combination of interleukin-2 and interferon-β. *Proc. Jpn. Acad.*, **62**, ser. B, 275.

IIGO, M., SAKURAI, M., TAMURA, T., SAIJO, N. & HOSHI, A. (1988). *In vivo* antitumor activity of multiple injections of recombinant interleukin 2, alone and in combination with three different types of recombinant interferon, on various syngeneic murine tumors. *Cancer Res.*, **48**, 260.

LANIER, L.L., BUCH, D.W., RHODES, L. and 4 others (1988). Interleukin 2 activation of natural killer cells rapidly induces the expression and phosphorylation of the Leu-23 activation antigen. *J. Exp. Med.*, **167**, 1572.

LOWENTHAL, J.W., RANSOM, J., HOWARD, M. & ZLOTNIK, A. (1988). Up-regulation of interleukin 4 receptor expression on immature (Lyt-$2^-$/L3T$4^-$) thymocytes. *J. Immunol.*, **140**, 474.

LUGO, J.P., KRISHNAN, S.N., SAILOR, R.D., KOEN, P., MALEK, T. & ROTHENBERG, E. (1985). Proliferation of thymic stem cells with and without receptors for interleukin-2; implications for intrathymic antigen recognition. *J. Exp. Med.*, **161**, 1048.

MATHIESON, B.J. & FOWLKES, B.J. (1984). Cell surface antigen expression on thymocytes: development and phenotypic differentiation of intrathymic subsets. *Immunol. Rev.*, **82**, 141.

METCALF, D. (1966). The thymus. *Recent Results Cancer Res.*, **5**, 43.

MORGAN, D.A., RUSCETTI, F.W. & GALLO, R. (1976). Selective *in vitro* growth of T lymphocytes from normal human bone marrows. *Science*, **193**, 1007.

PIGUET, P.F., GRAU, G., IRLE, C. & VASSALLI, P. (1986). Administration of recombinant interleukin 2 to mice enhances production of hemopoietic and natural killer cells. *Eur. J. Immunol.*, **16**, 1257.

RAULET, D.H. (1985). Expression and function of interleukin-2 receptors on immature thymocytes. *Nature*, **314**, 101.

ROSENBERG, S.A., SCHWARZ, S.L. & SPIESS, P.J. (1988). Combination immunotherapy for cancer: synergistic antitumor interactions of interleukin-2, alfa interferon, and tumor-infiltrating lymphocytes. *J. Natl Cancer Inst.*, **80**, 1393.

SMITH, K.A. (1980). T-cell growth factor. *Immunol. Rev.*, **51**, 337.